# Management of intrusive luxation with immediate surgical repositioning

**DOI:** 10.4103/0972-0707.55621

**Published:** 2009

**Authors:** Dibyendu Mazumdar, Pradip Roy, Pardeep Kumar

**Affiliations:** Department of Conservative Dentistry and Endodontics, Dr. R. Ahmed Dental College and Hospital, Kolkata, India

**Keywords:** Intrusive luxation, splint, surgical extrusion

## Abstract

Intrusive luxation is one of the most severe forms of traumatic injuries in which the affected tooth is forced to displace deeper into the alveolus. As a consequence of this type of injury, maximum damage occurs to the pulp and all the supporting structures. This report presents a case of severe intrusive luxation of mature maxillary central and lateral incisor in a 40-year-old male. The intruded tooth was immediately repositioned (surgical extrusion) and splinted within hours following injury. Antibiotic therapy was initiated at the time of repositioning and maintained for 5 days. Pulp removal and calcium hydroxide treatment of the root canal was carried out after repositioning. Splint was removed 2 months later. Definitive root canal treatment with Gutta percha was accomplished at a later appointment. Clinical and radiographic examination 6, 12 and 24 months after the surgical extrusion revealed satisfactory progressive apical and periodontal healing.

## INTRODUCTION

Luxation lesions account for 15.0% to 61.0% of traumas in permanent teeth.[1] Intrusion injury has a rarer occurrence in permanent dentition when compared with other types of luxation injuries. It comprises 3% of all traumatic injuries in the permanent dentition[2] and 5%–12% of dental luxations.[3,4] Pulp necrosis, inflammatory root resorption, ankylosis, loss of marginal bone support, pulp canal obliteration, paralysis or disturbance of radicular development and gingival retraction may occur as a consequence of an intrusive luxation.[5,6,7] This case report discusses the management of traumatic injury in which three permanent teeth, including two central incisors and one lateral incisor, were severely intruded.

## CASE REPORT

**Figure 1:**
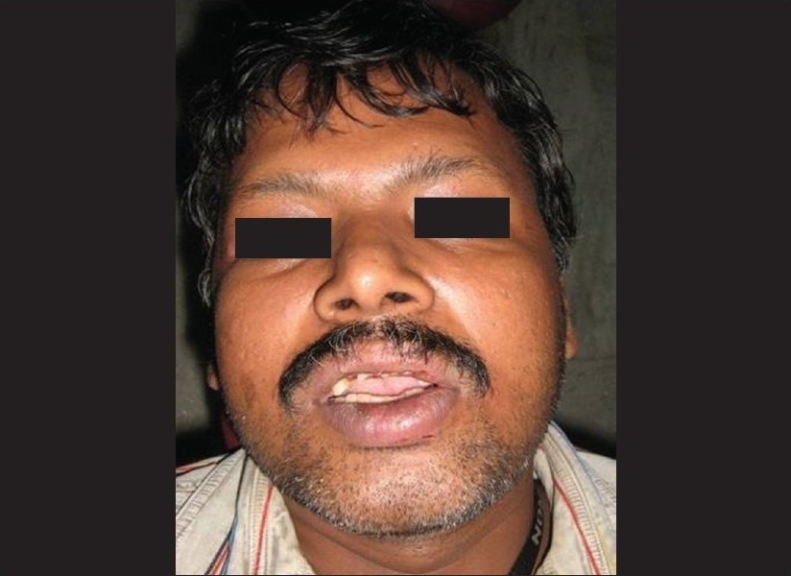
Pre-operative front view of patient immediately after trauma

**Figure 2:**
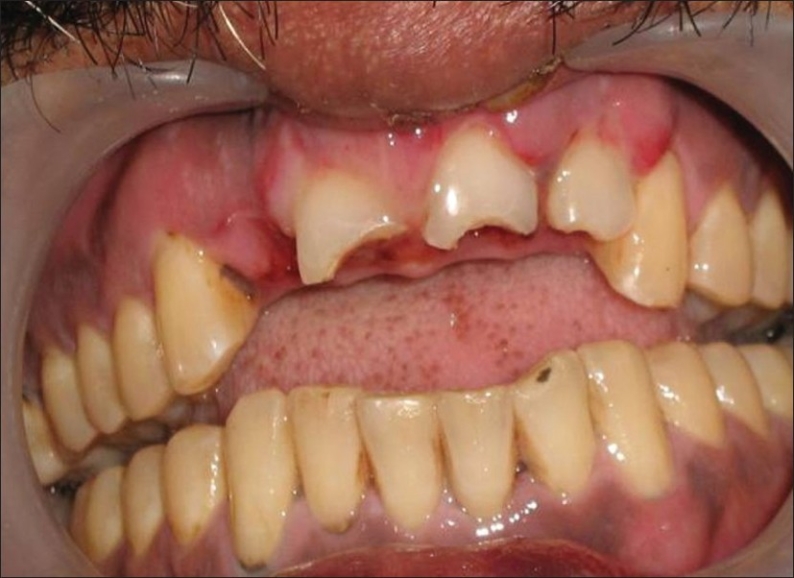
Intra-oral view showing intruded upper central incisors and left lateral incisors, with loss of upper right lateral incisor

**Figure 3:**
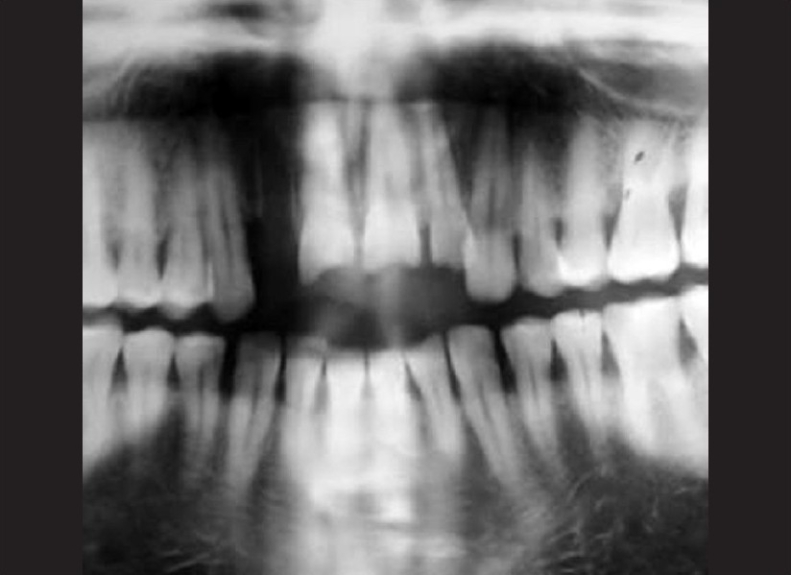
Orthopantomograph of the patient showing the intruded upper incisors with their abnormal relative position with other teeth

The patient was a healthy, 42-year-old male [Figure 1] who had suffered a roadside accident 2 h before and after initial examination and soft tissue management, he was referred to the conservative Dentistry PG Clinics of Dr. R. Ahmed Dental College and Hospital by Oral Surgery Department of the same college Clinical examination revealed that the injury had resulted in 5–6 mm intrusion [Figure 2] and uncomplicated crown fracture of maxillary permanent central incisors and left lateral incisor [Figure 3].

Subluxation of both maxillary canines was also noted. There was additional injury to alveolar bone, teeth and surrounding soft tissues with minor lacerations on both upper and lower lips. Lower central incisors were also mobile due to trauma.

**Figure 4:**
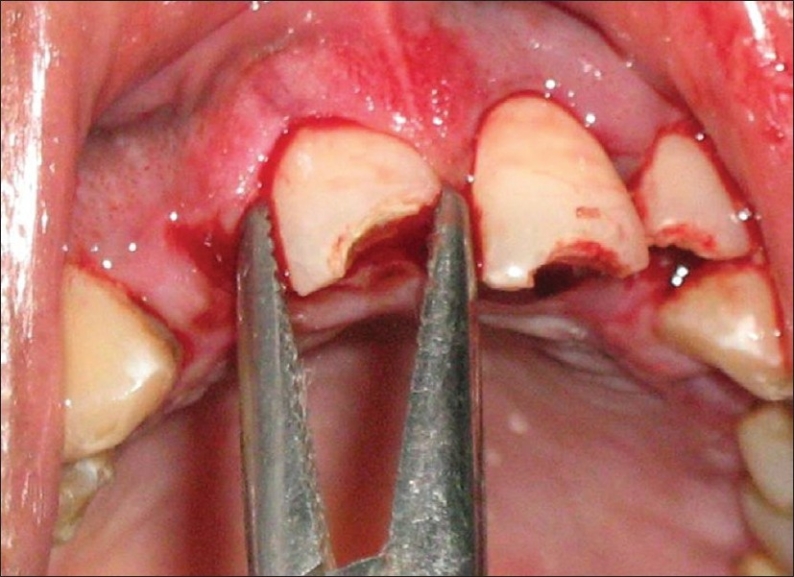
An artery forcep used to apply a very gentle downward force to position the teeth properly

**Figure 5:**
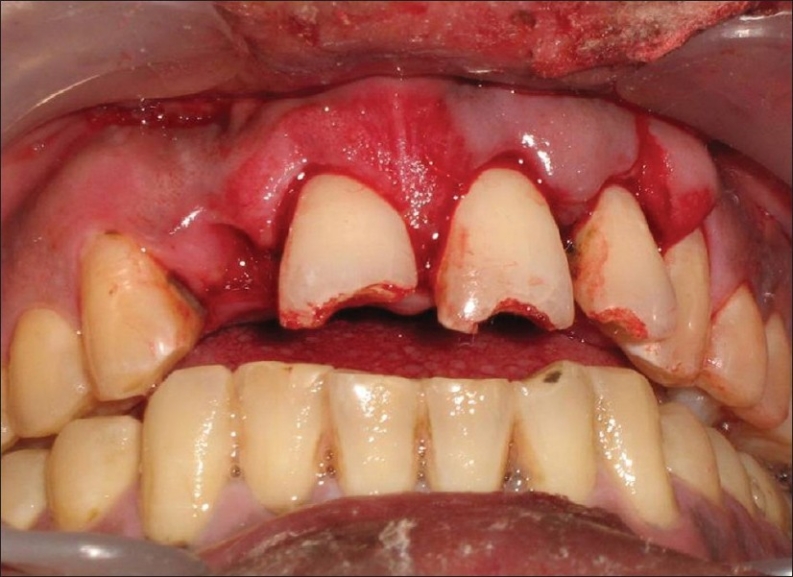
Space was present between the upper and lower teeth. Patient gave history of an pre-existing open-bite

**Figure 6:**
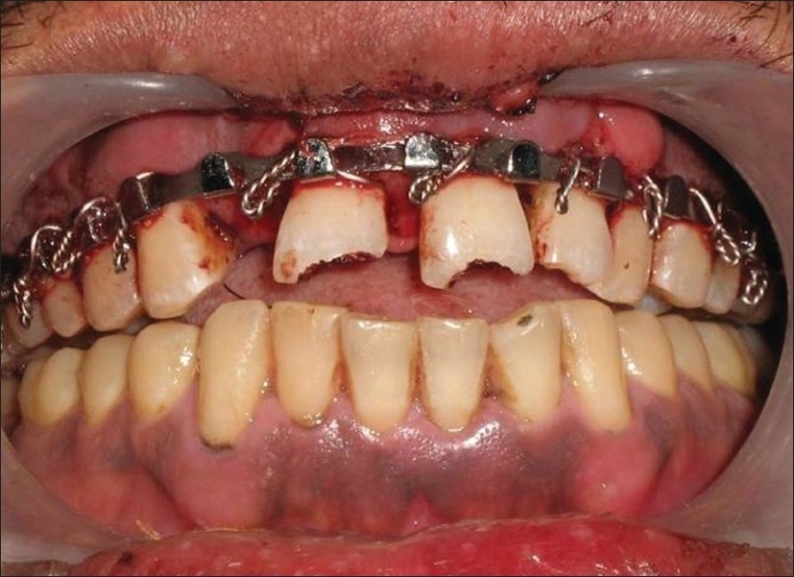
Splinting done using Erich‘s arch bar technique

Because of the severity of intrusion and completed root development, immediate surgical repositioning of intruded incisors was planned. Prior to surgical operation, the patient was given doxycycline (100 mg, oral). After the administration of local anesthesia, the intruded tooth was initially luxated. An artery forcep was used for this purpose [Figure 4]. The tooth was brought into a position by applying careful and very gentle force incisally. The teeth were repositioned to a level such that the cementoenamel junction (CEJ) was in plane with the free gingival margin. Following repositioning of the teeth, space was found to be present between the upper and lower teeth. Patient′s history of pre-existing open-bite justified the relative positions [Figure 5]. After bringing the upper lateral incisor and central incisors into their respective positions, these teeth were splinted using Erich's arch bar technique [Figure 6]. The lower incisors with slight mobility were splinted using 22 gauge wire and light-cured composite resin. Arch bar was used in the upper teeth because of the * associated alveolar fractures and extreme mobility* of the teeth involved in intrusion and mobility of both the canines as well. Another reason for avoiding composite splint in upper teeth was the continued bleeding from the traumatized supporting structures in upper teeth.

Upper left lateral incisor (tooth no. 22) was symptomatic 3 weeks later with patient complaining of pain and sensitivity in the same. Intra oral periapical radiograph (IOPA) showed external root resorption with loss of marginal bone support. Therefore, access was done in tooth no. 22, and the necrosed pulp was removed. Calcium hydroxide was placed as an intracanal medicament.

Splints were removed after 6 weeks. Both upper and lower central incisors were stable, while the upper left lateral incisor was found to be slightly mobile. Soft tissue injuries had healed. Therefore, continued need for support of tooth no. 22 was addressed to and a resin composite-wire splint was placed for 2 more weeks.

Follow-up IOPA radiograph showed narrowing of root canal lumen of upper central incisors and no response to pulp vitality tests. Hence a diagnosis of non-vital pulp was made and root canal therapy was completed.

**Figure 7:**
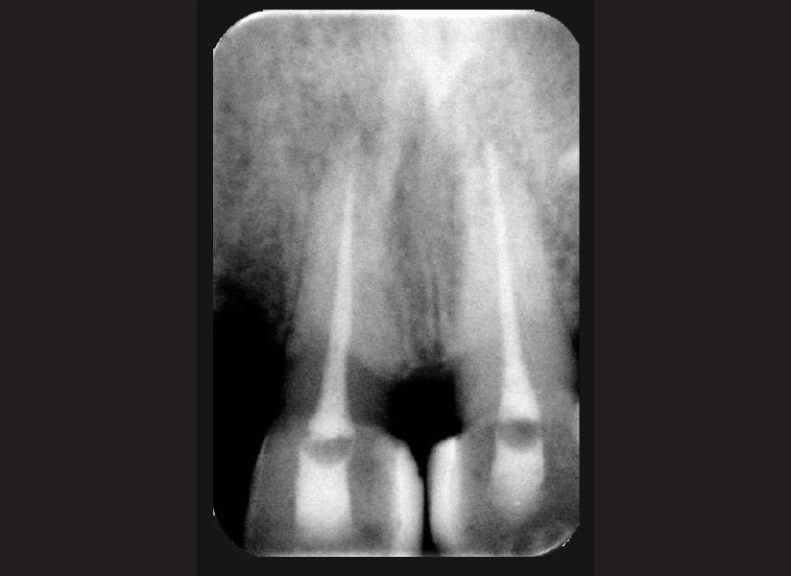
IOPA radiograph at 1-year follow up

**Figure 8:**
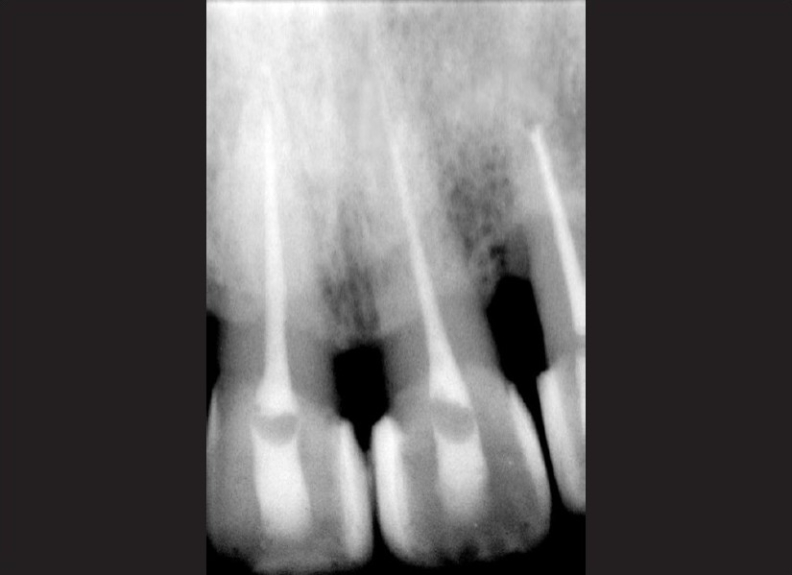
IOPA radiograph at 2-year follow up

Fractured incisal edges of both central incisors were built up with resin composite. Teeth were stable and patient was completely asymptomatic 6 months post-operatively. Further follow up at 1 year and 2 year post-operatively showed no changes in asymptomatic status of the patient clinically. Radiograph of the teeth involved was taken, and the peri-apical area shows normal healing [Figures 7 and 8].

## DISCUSSION

Intrusion injuries have the poorest prognosis and complex treatment among all tooth injuries. No consensus has been reached on the optimal treatment of this type of injuries. The recommended treatment options for intruded teeth include the following (3): Allowing spontaneous re-eruption of the teeth. Immediate surgical repositioning and fixation. Orthodontic repositioning (extrusion).

The aim of the treatment was to restore the tooth to its original position by decompressing the injured tissues and re-establishing the normal relationship between bone and tooth.

Andreasen and Andreasen[1] postulated that fully mature intruded teeth must be extruded by orthodontic means over a 2–3 week period. According to them, total repositioning by surgical means in such cases may increase the risk of resorption. However, their study is based on a pooled sample of different injury types, including subluxation, extrusion and intrusion. The unique nature of the intrusion injury distinguishes it from other luxations.

In addition, many previous studies, including that of 29 intruded permanent teeth by Kinirons, Sutcliff and Ebeleseder * et al.,* failed to show that surgical repositioning increased the prevalence of resorption.

In the presented case, immediate surgical repositioning was used for treating intruded incisors with favorable results at 2-year follow up. Still further follow up is continued to obtain a better interpretation of the result.

There are many advantages of this surgical technique for managing intruded teeth. First, it can be easily repositioned and provides the original anatomic situation for healing of the adjacent tissues and also provides adequate room for endodontic access. However, inadvertent ex-articulation during the repositioning procedure and possible additional damage to the periodontal ligament, leading to a higher risk of ankylosis, are disadvantages of the surgical procedure, which perhaps can be minimized depending on the caution and the skill of the operator.

The elapsed time for repositioning of the tooth was approximately 2 h in the presented case, which is an unfavourable factor, according to Andearsen delay in mechanical repositioning, more than 90 min facilitated replacement root resorption in the intruded position. In this case, more complications occurred in the lateral incisor, which may be correlated with severe injury encountered by this particular tooth.

## CONCLUSION

Pulp tissue and periodontal structures require constant attention in intrusive injuries. The treatment has to be adaptable according to complications. Further surgical repositioning in the presented case with 2-year follow up proved to be a viable treatment method for intruded teeth without any additional risk of resorption. The occurrence of such complications seems to be related to the degree of severity of the original injury. Further interpretation of results requires continued long-term follow up of the case.
